# DNA N6-Methyladenosine modification role in transmitted variations from genomic DNA to RNA in *Herrania umbratica*

**DOI:** 10.1186/s12864-019-5776-0

**Published:** 2019-06-18

**Authors:** Mei-Wei Luan, Wei Chen, Jian-Feng Xing, Chuan-Le Xiao, Ying Chen, Shang-Qian Xie

**Affiliations:** 1 Research Center for Terrestrial Biodiversity of the South China Sea, Hainan Key Laboratory for Biology of Tropical Ornamental Plant Germplasm, Natural Rubber Cooperative Innovation Centre of Hainan Province & Ministry of Education of China, Hainan University, Haikou, 570228 China; 2 State Key Laboratory of Ophthalmology, Zhongshan Ophthalmic Center, Sun Yat-sen University, Guangzhou, 510060 China

**Keywords:** 6mA modification, DNA variation, RNA variation, Transmitted variation

## Abstract

**Background:**

DNA methylation is an important epigenetic modification. Recently the developed single-molecule real-time (SMRT) sequencing technology provided an efficient way to detect DNA N6-methyladenine (6mA) modification that played an important role in epigenetic and positively regulated gene expression. In addition, the gene expression was also regulated by genetic variation. However, the relationship between DNA 6mA modification and variation is still unknown.

**Results:**

We collected the SMRT long-reads DNA, Illumina short reads DNA and RNA datasets from the young leaves of *Herrania umbratica*, and used them to detect 35,654 6mA modification sites, 829,894 DNA variations and 60,672 RNA variations respectively, among which, there are 303 DNA variations and 19 RNA variations with 6mA modification, and 57,468 transmitted genetic variations from DNA to RNA. The results illustrated that the genes with 6mA modification were significant disadvantage to mutate than those genes without modification (*p*-value< 4.9e-08). And result from the linear regression model showed the 6mA densities of genes were associated with the transmitted variations type 0/1 to 1/1 (*p*-value < 0.001).

**Conclusions:**

The variations of DNA and RNA in genes with 6mA modification were significant less than those in unmodified genes. Furthermore, the variations in 6mA modified genes were easily transmitted from DNA to RNA, especially the transmitted variation from DNA heterozygote to RNA homozygote.

**Electronic supplementary material:**

The online version of this article (10.1186/s12864-019-5776-0) contains supplementary material, which is available to authorized users.

## Introduction

N6-methyladenine DNA modifications (6mA) refers to the DNA adenosine based with a methyl group at the nitrogen-6 position, which differed from the 5-Methylcytosine (5mC) that methylated on the carbon− 5 position of Cytosine and extensively studied in eukaryotes. DNA 6mA modifications were initially discovered and commonly characterized in prokaryotes, involving gene expression, DNA replication and repair, and host-pathogen interactions [1, 2]. Recently, the existence of 6mA DNA methylation has also been detected and confirmed in eukaryotes, such as fungi, plants, animals and even mammalian [3, 4]. The global expression and feature profiling of DNA 6mA modifications revealed that they played important roles in regulating gene expression, embryonic development, and tumor genesis in *Arabidopsis thaliana*, *Mus musculus* and *Homo sapiens* respectively [5–7]. The developed single-molecule real-time (SMRT) sequencing technology provided a high-resolution strategy to identify 6mA modification based by the specific inter-pulse duration (IPD) signal during DNA synthesis [8–12]. Due to the advantage of DNA 6mA modification at the single-nucleotide resolution, it has got more attention and extensive detection in eukaryote [7, 13, 14].

The levels of gene expression were associated with genetic variation, and the variations contributed to gene expression are known as expression quantitative trait loci (eQTL) or single qualitative locus [15]. The eQTL that regulated gene expression has been reported in many species [16], such as maize [17], rice [18], and pig [19]. Besides, the genetic variation associated with the expression of specified gene had also been investigated. The allelic variation on ACD6 strongly increased gene expression in *Arabidopsis thaliana* [20]. Natural genetic variations that associated with gene expression were ubiquitous in diverse organisms including plants and animals [21]. In addition, the relationship between genetic variations and DNA 5mC methylation levels were studied in human brain and oil palm [22, 23]. Recently, some researchers have also found that gene expression was reinforced by DNA 6mA modifications in *Arabidopsis thaliana*, *Mus musculus* and *Homo sapiens* [5, 24, 25]. However, the relationship between genetic variation and DNA 6mA modification is still unknown.

In this study, we explored the relationship between DNA 6mA modifications and DNA and RNA variations by using the sequencing datasets from the young leaves of *Herrania umbratica*, which is a species of flowering plant from the family Malvaceae. The DNA 6mA modification, DNA and RNA variations were detected by using SMRT long-reads and Illumina short reads DNA and RNA datasets respectively. Furthermore, we decoded the global distribution patterns of 6mA in *H. umbratica*, and analyzed the role of DNA 6mA modification in the transmitted variations from DNA to RNA in *H. umbratica*. The results indicated that the variation ratio of 6mA modified genes was significant less than unmodified genes, and the variations in 6mA modified genes were easily transmitted from DNA to RNA, especially the transmitted variation from DNA heterozygote to RNA homozygote.

## Results

### Characterization of 6mA modification in *H. umbratica* genome

**Fig. 1:**
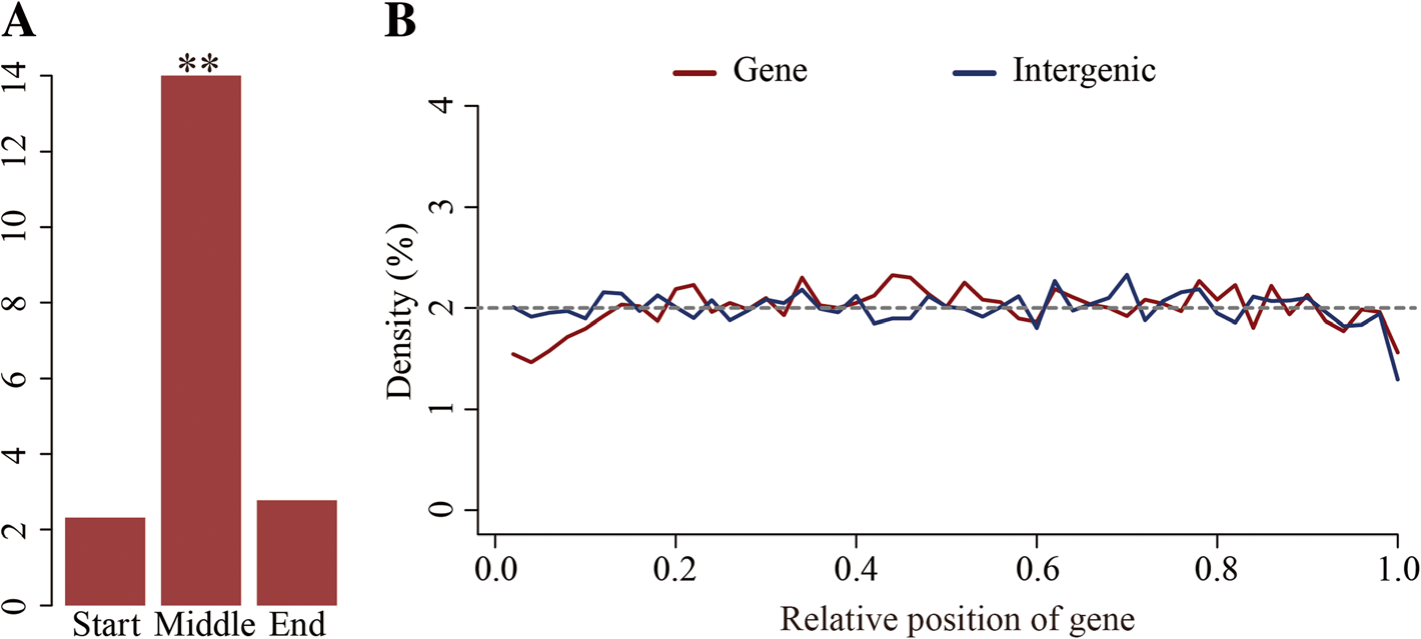
Density of 6mA modification in exon of *H. umbratica* genome. **a** The density of 6mA in three groups: start is the first exon; end is the last exon and middle is the exons between first and last. **b** The density of 6mA modification at relative positions of gene and intergenic regions. The abscissa axis refers to the related position of 6mA in genes

The 35,654 6mA modification sites were identified from PacBio SMRT long-reads data [12]. The 6mA density that refers to the number of adenine (A) site with 6mA modification divide by the total number of A base (6mA/A) was about 0.048% in the *H. umbratica* genomic DNA. Different densities of 6mA modification were shown among 6074 scaffolds of genome, and NW_018397657.1 was with the highest density (11 6mA sites, density = 0.04545) (Additional file 1: Table S1). There were 20,040 genes distributing in scaffolds. The 6mA distribution density in all gene regions (0.049%) was higher than that in intergenic regions (0.025%). Among all annotated genes, there were 7049 genes with 6mA modification, and the gene LOC110428865 had the highest density (0.051) (Additional file 1: Table S2). To further understand 6mA distribution in genes, we evaluated the 6mA sites in every exon. According to different numbers of exon in gene, we divided the exons into three groups, which are start, middle and end against the location of the first, middle exon in gene. The density of 6mA modification in each group was significant different and the low density was in the start and the end exons (Fig. 1a). Furthermore, we analyzed 6mA density of relative position in each gene and intergenic region. The result showed that the density of 6mA was low in the end of gene and intergenic region (Fig. 1b).

**Fig. 2:**
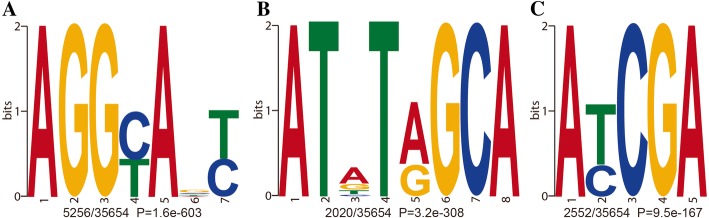
The consensus motifs of 6mA modification sites identified by DREME. (The 20 bp sequences upstream and downstream of 6mA site were used to detect motif. The number of occurrences of each motif relative to the total number of 6mA-containing motifs and the corresponding *P*-value were calculated by DREME. A, B and C referred to AGGYANY, ATNTRGCA and AYCGA motif sequence, respectively)

The conserved DNA sequence motifs of short nucleotides were widespread around 6mA modification sites [6]. To confirm the motif pattern in *H. umbratica*, we searched the significant enrichment of consensus motifs using DREME [26]. The 61 significant motif sequences that covered 30,055 6mA modification sites and accounted for 84.29% (30,055/35,654) were detected in *H. umbratica* (Additional file 1: Table S3). The AGGYANY, ATNTRGCA and AYCGA were the three most significant enrichment sequences of 6mA sites (Fig. 2). And AGGYANY motif accounted for 15% (5256) of all modification sites (35,654) (Fig. 2a, Additional file 1: Table S3), which is consistent with the AGG 6mA motif sequence in *C. elegans* [13], *O. sativa* [27], *A. thaliana* [5], and *H. sapiens* [6]. In addition, other enrichment of adenine (A) was evident in the motif sequence patterns ATNTRGCA and AYCGA (Fig. 2b and c). The results suggested that the conserved motif pattern of 6mA sites existed in the *H. umbratica* genome.

### Distribution of DNA and RNA variations

**Fig. 3:**
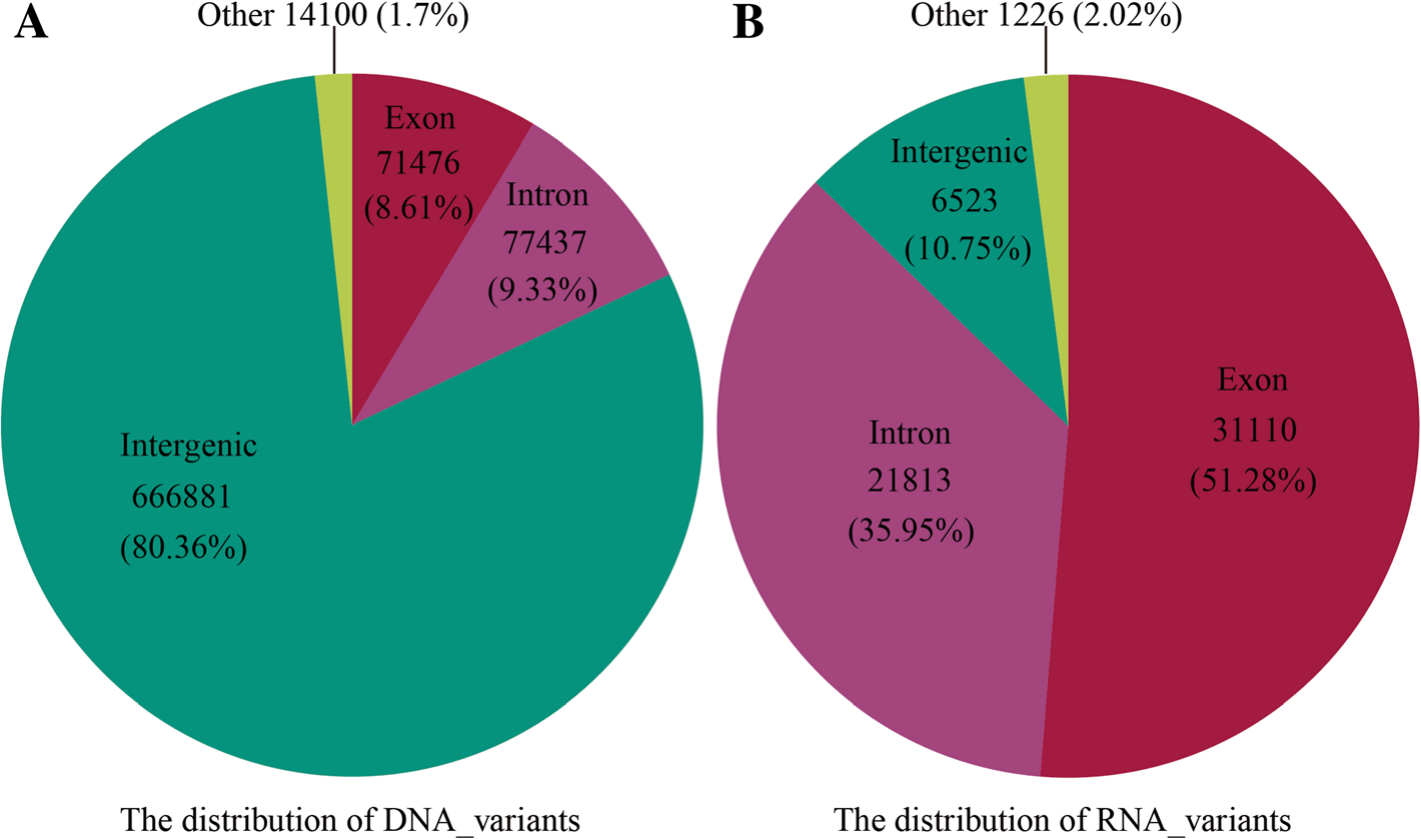
Distribution of DNA and RNA variations in *H. umbratica*. **a** DNA variations distribution. **b** RNA variations distribution

There were 829,894 variations detected in *H. umbratica* genomic DNA along with 4139 scaffolds (Additional file 1: Table S4), and 44,295 of them was in NW_018397254.1 with the most mutations. Among these variations of DNA, 163,013 (20%) occurred in 15,021 genes and the others in intergenic regions (Fig. 3a and Additional file 1: Table S5), and the highest variation frequency gene is LOC110428915 (density = 0.1771). There were 80% DNA variations appeared on intergeic regions, which might be caused by the length of intergenic regions (153,918,277) was twice longer than that in genes. In addition, 71,476 of 163,013 variations were on exons and 77,437 were on introns (Fig. 3a). In the distribution of RNA variations, we found that 60,672 variations were located in 135 scaffolds, and the most variations were detected in scaffolds NW_018397254.1 (6512 variations) (Additional file 1: Table S6). The 54,149 (89%) variations of 60,672 occurred in 12,255 genes (Additional file 1: Table S7), and more than half of RNA variation (51.28%) is in exons (Fig. 3b), which may be caused by the alternative splicing during the transcription from DNA to RNA in biological central dogma.

### Correlation between DNA 6mA modification and DNA/RNA variations

**Fig. 4:**
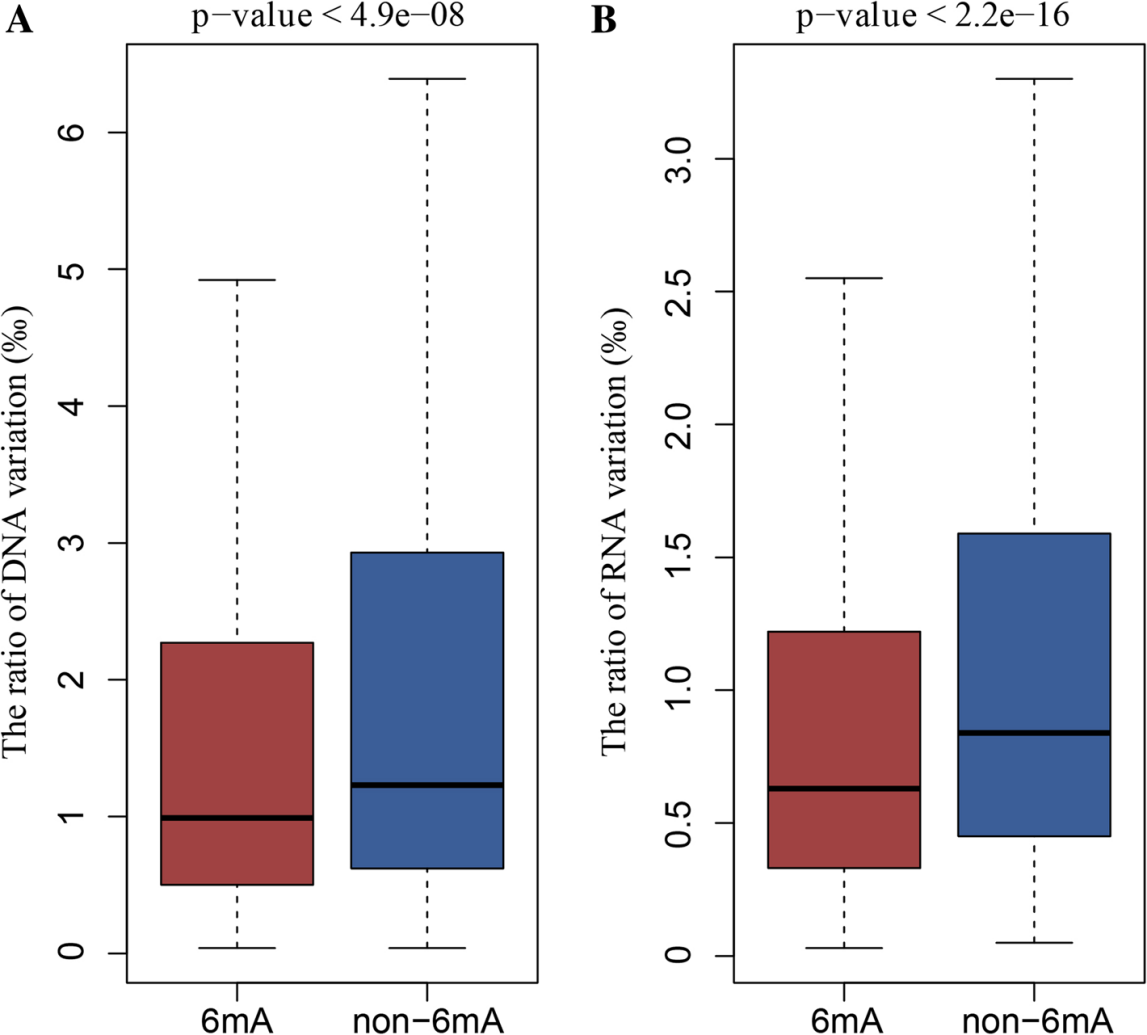
Comparison of variation ration in gene with and without 6mA modification. 6mA and non-6mA were the gene with 6mA modification and without 6mA modification. **a** Ratio of DNA variations between gene with and without 6mA modification. **b** Ratio of RNA variations between gene with and without 6mA modification

To further understand the relationship between DNA 6mA modification and DNA/RNA variations, we analyzed the feature of 6mA and variation sites of DNA and RNA. There were 303 sites which are both with DNA 6mA modification and DNA mutation, and 27 of which were in exons, 26 in introns, 7 in pseudogenes and 243 in intergenic regions (Additional file 1: Table S8). The number of common modification and mutation sites was obvious less than the total 6mA modification number (35,654) and DNA variation sites number (829,894), which just accounted for 0.85 and 0.04% respectively. We further compared the ratio of DNA variations between genes with and without 6mA modifications. The result showed that the genes with 6mA modification were significant disadvantage to mutate than those genes without modification (*p*-value < 4.9e-08, Fig. 4a). Interestingly, we also found that the 6mA density of gene with variations was lower than these genes without variation (Additional file 2: Figure S1). All these results above indicate that the DNA 6mA modifications lower the tendency of gene mutation and promote genes stability.

For RNA variation, there were 19 concurrent sites between 6mA modification and RNA variation, and 11 of them were in exon regions (Additional file 1: Table S9). We also analyzed the relationship between 6mA modification and RNA variations in gene regions. The result showed that the proportion of RNA variation on genes with 6mA modification was significant lower than the genes without modifications (p-value < 2.2e-16, Fig. 4b), which had the similar conclusion as described in DNA.

### Role of 6mA modifications in transmitted variations from DNA to RNA

**Fig. 5:**
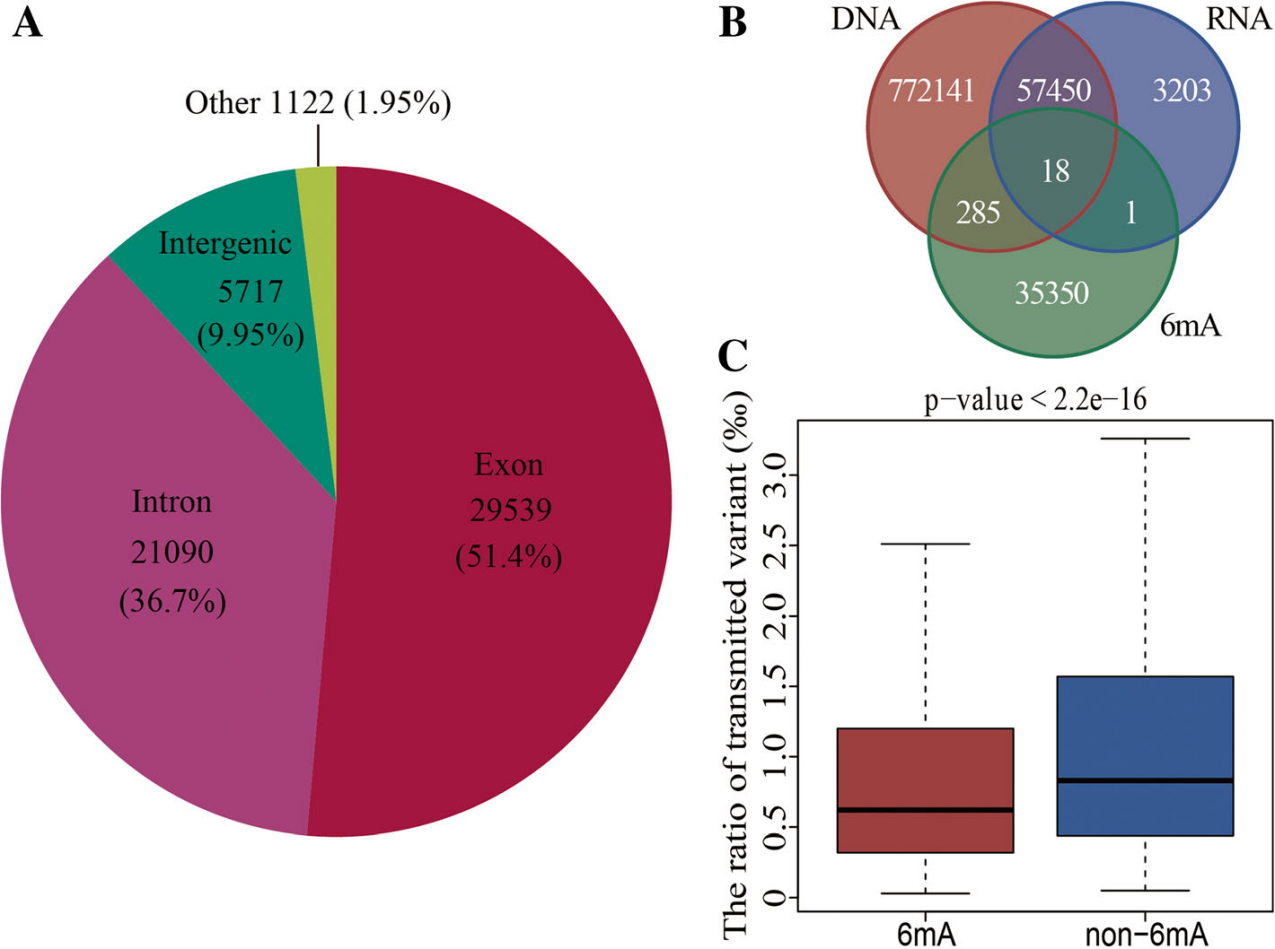
The role of 6mA modification in transmitted variations from DNA to RNA. **a** The distribution of transmitted variations in genome including exon, intron and intergenic regions. **b** Venn of variation sites and 6mA modification sites. Red, blue and green set were the DNA variations, RNA variations and 6mA modification sites. **c** The ratios of transmitted variations in genes with 6mA and unmodified

In order to understand the role of 6mA modifications in dynamic variations, we further analyzed the transmitted variations from DNA to RNA, including variations from DNA homozygous to RNA homozygous (1/1–0/0 and 1/1–1/1), homozygous to heterozygous (1/1–0/1), heterozygous to homozygous (0/1–0/0 and 0/1–1/1), and heterozygous to heterozygous (0/1–0/1), where 0 and 1 were normal and mutated base. The total 57,468 transmitted variation sites were detected and accounted for 6.92 and 94.72% of DNA (829,894) and RNA (60,672) respectively, among them, 29,539 of 57,468 were on exons and 21,090 on introns (Fig. 5a). Most of the RNA variations were transmitted variations, which suggested that the most mutations of RNA were from DNA variations. Besides, there were 19 RNA variations with 6mA modifications, and 18 of them were transmitted from DNA to RNA (Fig. 5b). Furthermore, the ratios of transmitted variations in genes with 6mA modification were less than unmodified genes (Fig. 5c). The results indicate that the 6mA modification is associated with transmitted variations.

To analysis the detail between 6mA density and variations transmitted from DNA to RNA, we established the linear regression model to fit the correlation between the 6mA densities of genes and the dynamic variations from DNA to RNA (Additional file 1: Table S10). The results of linear regression showed the 6mA densities of genes were associated with the transmitted variations type 0/1 to 1/1 (*p*-value < 0.001, Table 1), which suggested that the 6mA modifications affect the transmitted variations from DNA to RNA significantly. The mutated sites would be favorably transmitted from DNA to RNA in the genes with 6mA modification. The results demonstrated that 6mA modifications played a role in transmitted variations and might result in the different expression of gene.

**Table 1:** Regression analysis of 6mA density and transmitted variation from DNA to RNA in genes

Transmitted type	Estimate	Std.	Error	t_value
0/1_0/0	1.343e-05	1.024e-05	1.312	0.1895
0/1_0/1	-5.952e-06	2.418e-06	−2.462	0.0138*
0/1_1/1	4.476e-05	1.070e-05	4.184	2.89e-05***
1/1_0/0	−5.946e-04	1.045e-03	−0.569	0.5694
1/1_0/1	2.207e-04	6.025e-04	0.366	0.7141
1/1_1/1	5.740e-05	3.132e-05	1.833	0.0669

‘*’: significance level < 0.05; ‘***’: significance level < 0.0001

### Regulation of 6mA in intergenic regions

**Fig. 6:**
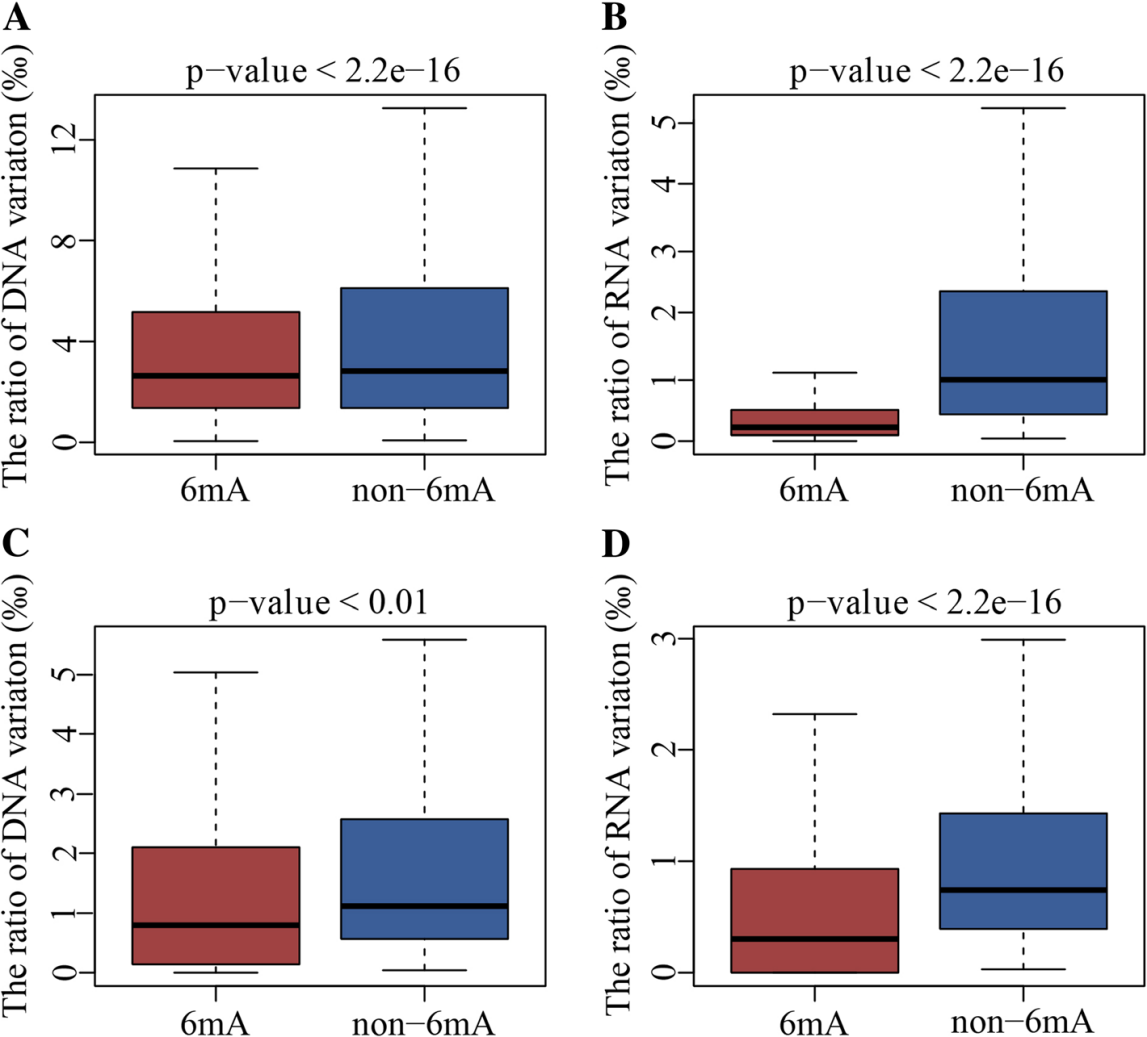
The relationship between transmitted variations and 6mA modification. **a** The ratios of DNA transmitted variations in intergenic regions with and without 6mA modification. **b** The ratios of RNA transmitted variations in intergenic regions with and without 6mA modification. **c** The ratios of DNA variations in genes with and without upstream modified intergenic regions. **d** The ratios of RNA variations in genes with and without upstream modified intergenic regions

The 6mA modification of genes played an important role in DNA, RNA and transmitted variations. Compared with annotated gene region, the most 6mA sites (65%) and variations (80% of DNA and 11% of RNA) were in the intergenic regions. To explore the relationship between 6mA modification and variation in intergenic regions, we compared the ratio of variations in intergenic regions with and without 6mA modification. The result illustrated that the ratio of variations in intergenic regions with 6mA modification was lower than those without 6mA sites (Fig. 6a and b). More importantly, we found the 6mA modification in intergenic region was associated with the mutation in downstream gene. If the 6mA modification was identified in the intergenic region upstream of gene, the mutation rate of the genes would be significant lower than that with normal upstream intergenic regions (Fig. 6c and d). The results demonstrated the 6mA modification in intergenic regions were disadvantage of mutation in downstream genes. Besides, we also found the similar result that the transmitted variation ratio in intergenic region with 6mA modification was less than unmodified region (Additional file 2: Figure S2).

## Discussion

N6-methyladenine (6mA) was one of the most important epigenetic marks in recent studies, which had been proposed to regulate gene expression and control numerous cellular and biological processes [1, 3, 5, 6, 13, 28, 29]. In this study, we firstly investigated the role of DNA 6mA modifications in genetic variations, including two aspects: (1) the association between 6mA modifications and DNA/RNA variations; (2) the role of 6mA modifications in transmitted variations from DNA to RNA. Interestingly, the DNA 6mA modifications trended to maintain the stability of DNA and RNA. Once the genes were with the high level of 6mA modifications, it would be difficult to mutate in DNA and RNA (Fig. 4). In addition, 6mA modification would be favorable to retain transmitted variations from DNA to RNA if gene was with 6mA modification (Table 1).

Most of 6mA modification sites and DNA variations were on intergenic regions of *H. umbratica*. We found that the upstream intergenic regions with 6mA modification leaded to the lower mutated ratio of downstream genes (Fig. 6), which implied the 6mA modifications in intergenic region may regulate the variation of downstream gene. The similar relationship also found that the transmitted variation ratio in intergenic region with 6mA modification was less than unmodified region (Additional file 2: Figure S2). These results illustrated that the DNA 6mA modifications regulated variations in intergenic regions and indirectly influenced the gene expression. Two regulatory factors containing splice acceptor and promoter in intergenic regions were important for the regulation of gene expression [30–32]. The role of 6mA modification in intergenic regions may indirectly affect the gene expression by two factors, which needed to be further studied and verified.

We collected SMRT long-reads and Illumina short reads datasets from the young leaves of *H. umbratica*. The specific-tissue contributed to different gene expressions [33, 34]. The DNA and RNA datasets used in this study were from the same tissue. Besides, we collected five RNA datasets from 5 different tissues including young leaves, open flowers, lateral buds, closed flower buds and apical stems, and analyzed the variations from different tissues. The gene expression of young leaves was significantly different from other tissue (Additional file 1: Table S11 and Additional file 2: Figure S3A). Total 73,976 SNPs in 5 tissues were detected, and the patterns of 5 tissues in exon and intron were also different (Additional file 2: Figure S3B). These results indicated that the RNA variations were different from five tissues in *H. umbratica*. Thus the variants detected from the same tissue used in this study were reasonable and reliable. Meanwhile, we calculated the abundance of gene expression from RNA-seq data in young leaves. And the expression of gene with 6mA modification was higher than unmodified gene (Additional file 2: Figure S3C). The 6mA modification playing a positive role in gene expression was consistent with previous studies [27, 28, 35].

To date, the potential epigenetic roles of 6mA in various eukaryotes has been well documented [3, 5, 6, 13]. The 6mA genomic distributions, consensus motifs, and association with transcription were relatively known in plants [5, 27]. However, the role of 6mA modification in DNA and RNA variations and transmitted variations from DNA to RNA in other species such as *Arabidopsis thaliana*, *Mus musculus* and *Homo sapiens* are still unclear. We have addressed the relationship among 6mA modification, DNA and RNA variation and demonstrated the DNA 6mA modification lowered the DNA and RNA variations by using the genomic and transcriptomic datasets from *H. umbratica* young leaves in this study. Then we will further investigate and validate the role of 6mA modification in DNA and RNA variations in other species.

## Conclusions

In this study, we firstly investigated the role of DNA 6mA modification in genetic variations. The variations of DNA and RNA in genes with DNA 6mA modification were significant less than those in unmodified genes. Furthermore, the variations in genes with 6mA modification were easily transmitted from DNA to RNA, especially the transmitted variation from DNA heterozygote to RNA homozygote.

## Materials and methods

### Data collection

We collected the long-reads DNA, short reads DNA and RNA sequence datasets of *Herrania umbratica* cultivar (Fairchild) from NCBI database with SRA accession SRP104516 (https://www.ncbi.nlm.nih.gov/sra/SRP104516). The long-reads DNA sequenced by SMRT PacBio RSII was used to detect DNA methylation of *H. umbratica* [12]. The short reads DNA and RNA sequenced by Illumina Hiseq platform were used for calling DNA and RNA variations respectively. All the 17 datasets of the young leaves tissue were collected in this study, including 12 PacBio long-reads DNA datasets (SRR5583222-SRR5583233), 4 short-read DNA datasets (SRR5580908, SRR5587191, SRR5605271, SRR5581326) and 1 RNA dataset (SRR5462401) (Table 2).

**Table 2:** Datasets collected in this study

Detection	RunID	Instrument	Size (G)	Seq_Type
DNA methylation	SRR5583222	PacBio RS II	3.6	DNA
	SRR5583223	PacBio RS II	3.5	DNA
	SRR5583224	PacBio RS II	3.2	DNA
	SRR5583225	PacBio RS II	2.6	DNA
	SRR5583226	PacBio RS II	2.9	DNA
	SRR5583227	PacBio RS II	2.6	DNA
	SRR5583228	PacBio RS II	12.2	DNA
	SRR5583229	PacBio RS II	11.4	DNA
	SRR5583230	PacBio RS II	11.2	DNA
	SRR5583231	PacBio RS II	7.9	DNA
	SRR5583232	PacBio RS II	4.2	DNA
	SRR5583233	PacBio RS II	3.9	DNA
DNA variations	SRR5580908	Illumina HiSeq 2500	15.4	DNA
	SRR5587191	Illumina HiSeq 2500	20.4	DNA
	SRR5605271	Illumina HiSeq 2500	18.2	DNA
	SRR5581326	Illumina HiSeq 2500	18	DNA
RNA variations	SRR5462401	Illumina HiSeq 2000	5.8	RNA

### Illumina DNA and RNA-seq data processing

The total 1,108,019,574 DNA raw short reads of *H. umbratica* were accessed and controlled quality by using FastQC v0.11.6 (FastQC: A Quality Control tool for High Throughput Sequence Data (https://www.bioinformatics.babraham.ac.uk/projects/ fastqc). The 167,310,955,674 sequence bases of *H. umbratica* were aligned to the reference genome (ASM216827v2) by using the BWA-MEM with default parameters [36, 37] (Additional file 1: Table S12). And the Picard tool (http://broadinstitute.github.io/picard) was used to sort reads and marked duplicate reads of the alignment bam file. Then we used the GATK Haplotypecaller to identify the genetic variations of DNA with parameters setting default [38, 39]. The variations calling and genotyping were performed across reference genome ASM216827v2. The indels of DNA variations was filtered by a custom Perl script, and the retaining SNPs were investigated in the following research.

The filtered 75,743,031 Illunima clean reads of RNA-seq were mapped to the *H. umbratica* reference genome (ASM216827v2) by Tophatv2.1.1 [40] (Additional file 1: Table S12), and Picard tool (http://broadinstitute.github.io/picard) was used to sort and mark duplicate reads for the bam file of mapped reads. Then the processing of RNA-seq variations calling and identifying were the same as described in the DNA processing [38, 39]. Finally, the variations VCF files of DNA and RNA data from the same sample were merged and compared by using BCFtools v1.7 (http://github.com/samtools/bcftools).

### Detection of DNA 6mA modification

The 12 flow cells of SMRT sequencing long-reads in h5 format were downloaded from the NCBI SRA database (Table 2). The overall 74x coverage of DNA long-reads were achieved against the *H. umbratica* genome, and the sequencing chemistry of SMRT long-reads was XL-C2. The PacBio SMRT analysis platform v2.3.0 (http://www.pacb.com/products-and-services/analytical-software/smrt-analysis/analysis-applications/epigenetics) was used to detect DNA 6mA modifications [12]. The detailed analysis workflows were as follows: Firstly, the raw reads were aligned to the reference genome by pbalign with the parameters ‘--seed=1 --minAccuracy=0.75 --minLength=50 --concordant --algorithmOptions=“-useQuality” --algorithmOptions=“-minMatch 12 -bestn 10 -minPctIdentity 70.0”’. Then the polymerase kinetics information was loaded after alignment by loadChemistry.py and loadPulses scripts with ‘-metrics DeletionQV, IPD, InsertionQV, PulseWidth, QualityValue, MergeQV, SubstitutionQV, DeletionTag’. Finally, the post-aligned datasets were sorted by using cmph5tools and the m6A was detected by using ipdSummary.py script with ‘--methylFraction --identify m6A --numWorkers 4’. 6mA sites with more than 25-fold coverage were retained for further analysis.

### Bioinformatics analysis

The genome-wide 6mA profiling across the annotated gene and intergenic regions was analyzed by using in-house shell scripts. For each 6mA modification site, we extracted 20 bp from the upstream and downstream sequences of the 6mA site respectively, and then used DREME to predict conserved motifs in the flanking regions [26]. The paired comparison analysis that compared the number of variations on DNAs and RNAs with 6mA modifications or normal, were tested by student *t*-test. To analyze the correlation between m6A modifications and transmitted variations from DNA to RNA, we used multivariate linear regression analysis. The linear model was *y*_*i*_*=* ${\sum}_{n=1}^6{a}_{in}{x}_{in}+\varepsilon$, where *y*_*i*_ was 6mA density of the *i*th gene, *a*_*in*_ and *x*_*in*_ refer to the regression coefficient and variation types number of the *i*th gene, and *ε* was residual term. Because the variations of each site in genes had three statuses: 0/0, 0/1, and 1/1, where 0 and 1 represented the normal and mutation, thus the transmitted variations from DNA to RNA had 6 types of variations (0/1–0/0, 0/1–0/1, 0/1–1/1, 1/1–0/0, 1/1–0/1, 1/1–1/1). Based on the linear model above, the significant transmitted variations type that associated with 6mA densities were selected and analyzed. Besides, we used R 3.1.0 to perform the statistical analysis and figures drawing in this study.

## Additional files

Additional file 1: **Table S1.** The 6mA densities of scaffolds. **Table S2.** The 6mA densities of genes. **Table S3.** The motif pattern in *H. umbratica*. **Table S4.** The number of DNA variants in scaffolds. **Table S5.** The number of DNA variants in genes. **Table S6.** The number of RNA variants in scaffolds. **Table S7.** The number of RNA variants in genes. **Table S8.** The DNA variations with 6mA modification. **Table S9.** The RNA variations with 6mA modification. **Table S10.** The number of variants transmitted from DNA to RNA in genes. **Table S11.** The t-test of gene expressions in five tissues. **Table S12.** Statistics of DNA and RNA reads. (XLSX 1370 kb)

Additional file 2: **Figure S1.** The 6mA density of genes with and without variation. **Figure S2.** The ratio of transmitted variants in intergenic regions with 6mA and without 6mA modification. **Figure S3.** The gene expression in different tissues of *H. umbratica*. (DOCX 369 kb)

## Abbreviations

5mC: 5-Methylcytosine
6mA: N6-methyladenine
A: Adenine
*A. thaliana*: *Arabidopsis thaliana*
BWA: Burrows Wheeler Aligner
*C. elegans*: *Caenorhabditis elegans*
DREME: Discriminative Regular Expression Motif Elicitation
eQTL: Expression quantitative trait loci
GATK: Genome Analysis Toolkit
*H. sapiens*: *Homo sapiens*
*H. umbratica*: *Herrania umbratica*
IPD: Inter-pulse duration
MEM: Maximal exact matches
NCBI: National Center for Biotechnology Information
*O. sativa*: *Oryza sativa*
SMRT: Single-molecule real-time
SNP: Single nucleotide polymorphism
SRA: Sequence Read Archive
