# Supplementary material for: DNA N6-Methyladenosine modification role in transmitted variations from genomic DNA to RNA in Herrania umbratica
Source: BMC Genomics. 2019 Jun 18;20:508. doi: 10.1186/s12864-019-5776-0 (PMC6582544; doi:10.1186/s12864-019-5776-0)
Supplement: Supplementary file 2 — Figure S1. The 6mA density of genes with and without variation. Figure S2. The ratio of transmitted variants in intergenic regions with 6mA and without 6mA modification. Figure S3. The gene expression in different tissues of H. umbratica. (DOCX 369 kb) [file 12864_2019_5776_MOESM2_ESM.docx]

**
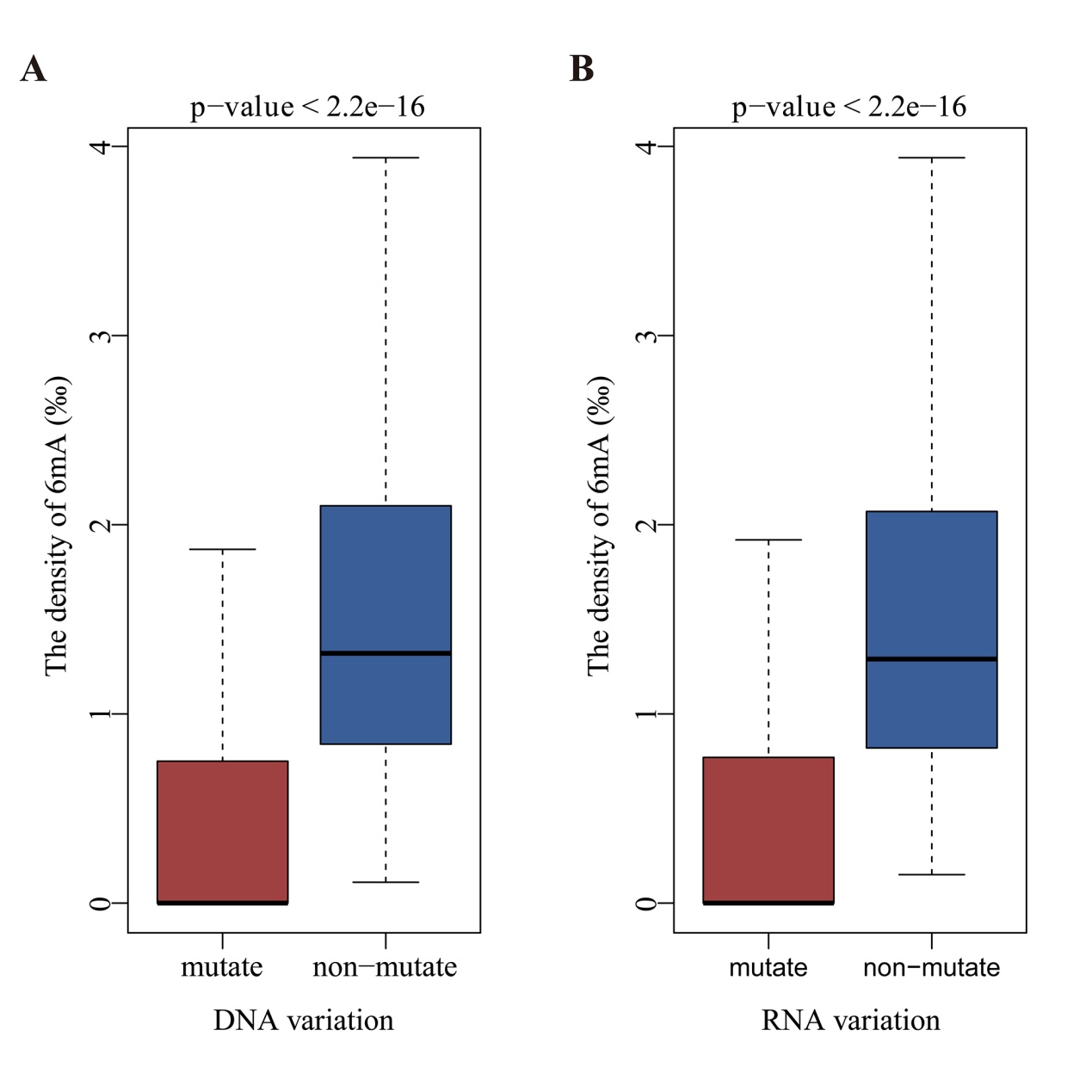
**

Figure S1. The 6mA density of genes with and without variation. Mutate and non-mutate are the gene with variation and without variation, respectively. (A) The 6mA density of genes with and without DNA variation. (B) The 6mA density of genes with and without RNA variation.

**
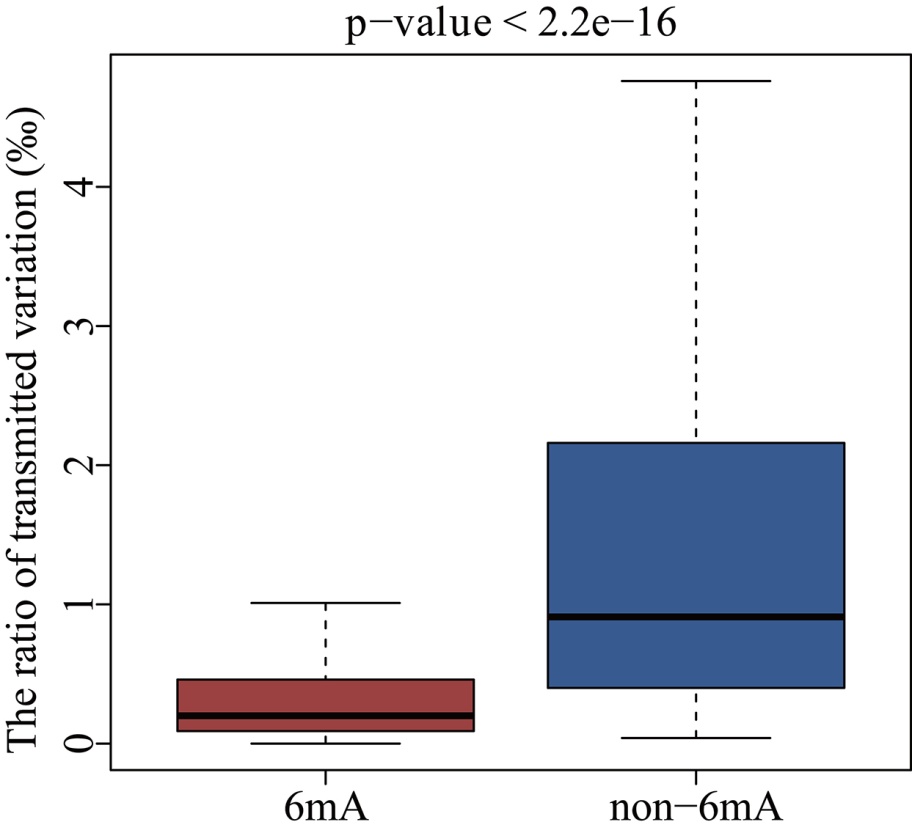
**

Figure S2. The ratio of transmitted variants in intergenic regions with 6mA and without 6mA modification.

**
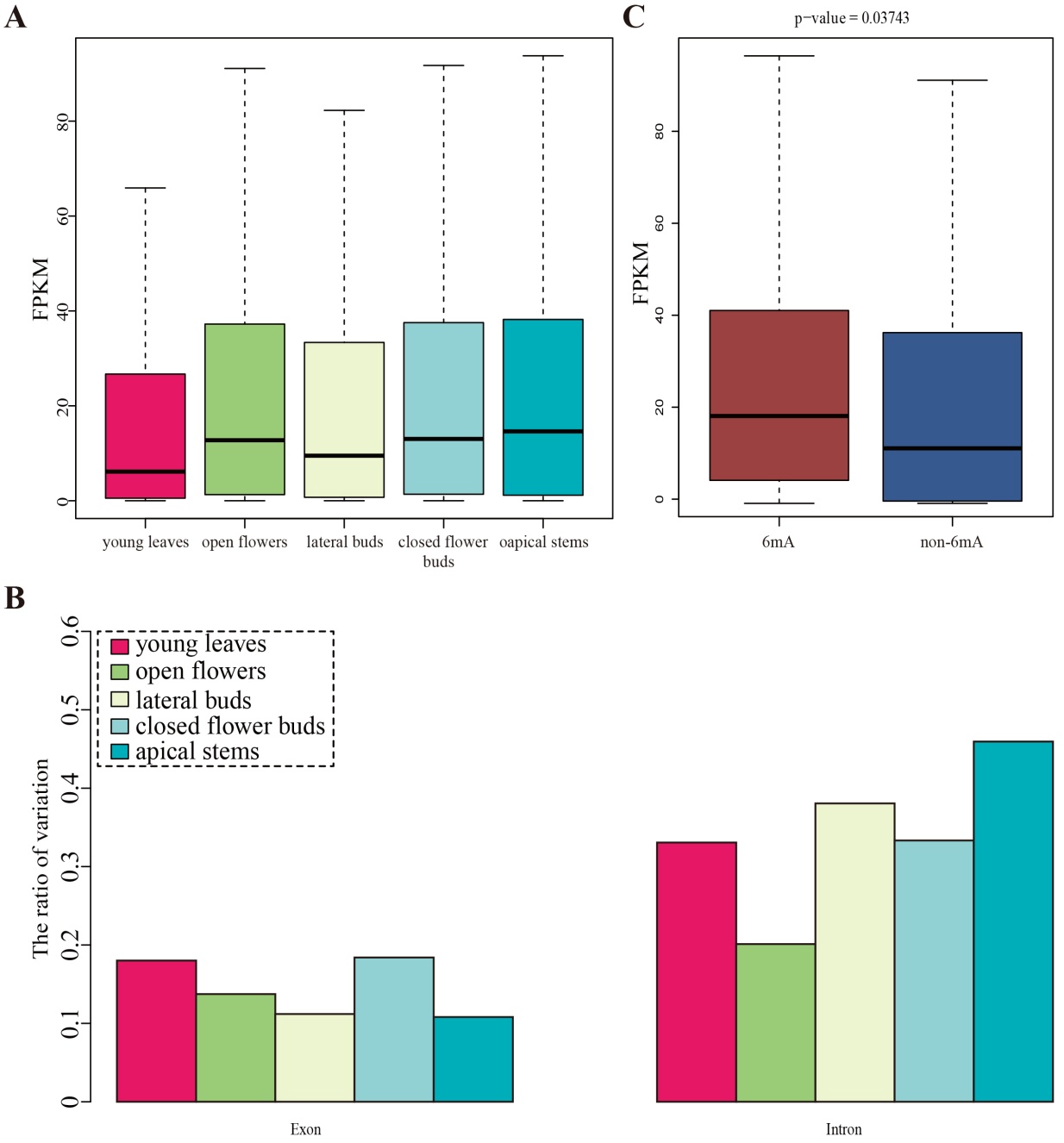
**

Figure S3. The gene expression in different tissues of *H. umbratica*. (A) The different gene expression in 5 tissues. (B) The ratios of tissue-specific variations in exonic and intronic region of 5 tissues. (C) The expression of gene with and without 6mA modification in young leaves.
